# Navigating Medical Device Certification: A Qualitative Exploration of Barriers and Enablers Amongst Innovators, Notified Bodies and Other Stakeholders

**DOI:** 10.1007/s43441-022-00463-4

**Published:** 2022-10-04

**Authors:** Rebecca Baines, Petra Hoogendoorn, Sebastian Stevens, Arunangsu Chatterjee, Liz Ashall-Payne, Tim Andrews, Simon Leigh

**Affiliations:** 1 The Organisation for the Review of Care and Health Applications (ORCHA), Daresbury, WA4 4AB UK; 2 University of Plymouth, Plymouth, PL4 8AA UK; 3 Leiden University Medical Center, Albinusdreef 2, 2333 ZA Leiden, Netherlands; 4 University of Leeds, Leeds, LS2 9JT UK; 5 Warwick Medical School, University of Warwick, Coventry, UK

**Keywords:** CE mark, Medical devices, Regulation, UKCA, Digital health

## Abstract

**Background:**

Medical device certification has undergone significant changes in recent years. However, exploration of stakeholder experiences remains relatively limited, particularly in the context of software as a medical device. This study sought to explore stakeholder experiences of medical device certification across both the UK and EU.

**Methods:**

Semi-structured interviews (*n* = 22) analysed using inductive-thematic analysis, synthesised using activity theory.

**Results:**

Innovators, consultants and notified bodies share more similarities than differences when discussing barriers and enablers to achieving medical device certification. Systemic tensions between existing rules, tools, community understanding and division of labour currently undermine the intended aim of certification processes. Existing rules are considered complex, with small and medium-sized enterprises considered disproportionality affected, resulting in several unintended outcomes including the perceived ‘killing’ of innovation. Existing certification processes are described as unfit for purpose, unethical and unsustainable.

**Conclusion:**

Stakeholder experiences suggest that the intention of establishing a robust and sustainable regulatory framework capable of ensuring a high level of safety whilst also supporting innovation is not yet being realised. Failure to enact desired changes may further jeopardise future innovations, outcomes and care quality.

**Supplementary Information:**

The online version contains supplementary material available at 10.1007/s43441-022-00463-4.

## Introduction

Digital health carries significant potential in making healthcare more affordable, accessible and effective [1–4]. The international use of medical device innovations has also seen a rapid increase in recent years [4–8], with start-ups and small-to-medium enterprises (SMEs) often frontrunners in producing such innovations [1, 9]. However, whilst advances in medical device innovation have resulted in both an increase in the number of medical devices, level of invasiveness and critical functionality [7], following a number of safety scandals i.e. the Poly Implant Prothèse (PIP) breast implant scandal [10–12], and metal on metal hip implants [7], medical devices can also present a number of challenges and risks [4, 13–15], with suggestions that the rapid growth of digital innovation has outpaced regulatory efforts [6].

The European Union’s Medical Device Regulation (EU MDR, Regulation 217/745) and other regulatory agencies such as the Food and Drug Administration (FDA, USA), The Medicines and Healthcare products Regulatory Agency (MHRA, UK) and Therapeutic Goods Administration (TGA, Australia) arguably recognise such concerns, stating that a “*fundamental revision”* of previous Directives “*is needed to establish a robust, transparent, predictable and sustainable regulatory framework for medical devices which ensures a high level of safety and health whilst supporting innovation”* ^(p.L117/1)^ [16]. The MDR states that *“this Regulation aims to ensure the smooth functioning of the internal market… taking as a base a high level of protection of health for patients and users and taking into account the small- and medium-sized enterprises that are active in this sector. At the same time, this Regulation sets high standards of quality and safety for medical devices in order to meet common safety concerns as regards such products”*^(p.L117/1)^ [16], highlighting its intended aim of supporting a smooth functioning market that supports SMEs within the digital health space, whilst simultaneously maintaining high standards of safety and quality.

The aims of EU regulations and others are to therefore set high standards of quality and safety for medical devices, with two new regulations (the MDR and In Vitro Diagnostic Device Regulation (IVDR)) set to replace current directives in a phased manner, with a 3-year transitional period for the MDR and 5-year transition for the IVDR [7]. However, whilst the implementation of the more stringent EU MDR is anticipated to lead to an increase in quality and “*strengthening and reinforcement”* of current systems [7], it may also be accompanied by a significant increase in cost and time to market [8, 17, 18], potentially overstretching start-ups’ capabilities [19]. However, exploration of whether such concerns have occurred across the digital innovation ecosystem is yet to be undertaken. In May 2021, The European Institute of Innovation and Technology (EIT) Health organized a virtual Focus Group to explore EU MDR readiness. One of the resulting recommendations was to assess the effects of the EU MDR on digital health specifically [20].

This research directly responds to the EIT recommendation by exploring how innovators, notified bodies and other stakeholders across the innovation ecosystem have experienced the medical device certification process, paying particular attention to how “fit for purpose” these regulations are, highlighting any barriers and where improvements could potentially be made.

## Methods

### Research Design

This research was based on a qualitative design which sought to understand stakeholders experiences, perceptions and suggestions for improvement with regard to the medical device certification process in the UK and EU. Justification for using a qualitative design stems from its ability to elicit opinions and perceptions of relatively unexplored areas [21] and provide rich insights into social processes, including peoples’ experiences and perceptions, to a greater extent than their quantitative counterparts. We utilised semi-structured interviews (*n* = 22) analysed via inductive-thematic analysis, a flexible method often used to generate a rich, yet detailed account of qualitative data, the discussion guides for which are provided in Supplementary File 1. These data were then synthesised using activity theory.

### Recruitment and Sampling

Participants from both the UK and EU were purposefully recruited on a voluntary basis via email, social media/network posts and personal invitations distributed via the personal and organisational networks of the research team and/or participants. Whilst this recruitment strategy may be subject to bias [22], a purposeful approach was selected due to the study’s focus on stakeholders’ experiences of medical device certification. All participants were given time to consider their involvement prior to participation.

### Data Collection

Semi-structured interviews were held online via Zoom or Microsoft Teams due to ongoing COVID-19 restrictions and participant locations. Three trained qualitative researchers [RB, SS, PH] conducted the interviews using a piloted interview schedule based on existing literature, with all interviews audio recorded with the verbal consent of participating individuals. Interviews lasted approximately 45–90 min. No repeat interviews were conducted. Interview data were transcribed verbatim by [RB], with any identifiable information replaced with relevant pseudonyms during the transcription process.

### Data Analysis

Data were initially analysed using inductive-thematic analysis as proposed by Clarke et al. [23]. Inductive-thematic analysis was selected for the purposes of this research as it is advocated as a useful and flexible method to generate a rich, yet detailed account of qualitative data. Analytical rigour was ensured by the independent coding of the interviews and scrutiny of suggested coding amongst two members of the research team [RB, PH] (Clarke et al. [23]). Participants were also offered the opportunity to provide feedback on initial findings to ensure relevance and accuracy, providing further analytical rigour.

### Data Synthesis

Activity theory was used as an interpretive framework to synthesise research findings [24]. Activity theory conceptualises any activity such as the process of applying for medical device certification as the interaction of six interdependent elements: (i) the *subject*, the individual or group from whose perspective the activity is being viewed; (ii) the *object,* the central issue at which the activity is directed and related *outcomes*, the purpose for which the *object* is used; (iii) the *tools*, which mediate between the subject and object outcomes; (iv) the *rules,* both explicit and implicit; (v) the *community*, multiple individuals or subgroups who share the same general object and (vi), the *division of labour*, the horizontal division of tasks between community members and the vertical division of power and status [24]. The description of activity theory as “*the best kept secret of academia”* ^(page 64)^ [24] and successful application in exploring successes, failures, and contradictions in complex situations including medical revalidation [25] without reductionist simplifications helps justify its inclusion in this research.

## Results

Twenty-two interviews (*n* = 9 SME innovators, *n* = 5 consultants, *n* = 3 NBs, *n* = 1 investor, *n* = 1 large innovator, *n* = 2 health authorities, *n* = 1 trade association) were conducted, providing a broad range of perspectives and experiences. Activity theory is used to synthesise findings beginning with the interdependent element of rules. The subject, or individual from whose perspective the activity is being viewed from is indicated by the participant identifier i.e. innovator, notified body, and is therefore not included as a separate section in the results write up to avoid duplication.

## Rules

### Complex and Uncertain

Whilst often recognised as being implemented in response to previously “*flawed processes”* (innovator 9) such as the “*breast implant scandal”* (innovator 9), many participants described the “*extremely long”* (consultant 3) and “*changing”* (notified body (NB hereafter) 3) certification rules as “*unclear”* (consultant 3), “*complex”* (innovator 3) and “*inconsistent”* (NB 2). Whilst a lack of clarity was at times described as being advantageous as “*you can build your own story that it’s a class I device”* (investor), “*the definition of medical device”* was repeatedly described as “*grey”,* with a “*lack of consistency and clarity”* resulting in a lack of “*awareness*” and “*confusion across the board”* (trade association). As a result, many innovators became aware of their products’ medical device status “*too late”* (NB 3): “*this is the first hurdle, knowing that it* [regulation] *exists and knowing you are a medical device, a non-trivial hurdle not anticipated by lots of manufacturers”* (NB 1). Many participants considered existing certification rules to be “*over detailed”* (NB 2), with limited “*harmonization”* (consultant 1) between “*regions”* (innovator 1), resulting in a regulatory “*minefield”* (innovator 2).

### Political Influences

Such confusion was at times attributed to unhelpful, “*bureaucratic”* (consultant 3) and political influences including “*the tumultuous Brexit split”* (innovator 2), with key stakeholders “*no longer at the table to make the law clear”* (large company). A perceived disparity between UK and “*European Union”* (NB 1) rules was also considered counterproductive “*for a lot of UK companies that want to access the EU markets”* (large company) and vice versa—“*it’s such a shame that the UK is out, that’s another hurdle because now I have to go for the MDR in the UK as well”* (innovator 3).

### Unclear Definitions of Significant Change and ‘Sufficient’ Clinical Evidence

Areas of particular confusion included defining a “*significant change”* (health authority 2) and “*sufficient”* clinical evidence. For example, when asked ‘when is clinical evidence sufficient enough?’, one consultant replied, “*no one knows… there’s no agreement on what clinical evidence is under the MDR”* (consultant 3). For other participants, “*it depends”* (NB 1) was a more common response, often based on the “*intended purpose or class of the device”* (NB 1). Similar responses were also provided when asked ‘what is currently considered a significant change?’, “*that depends, per device, per change”* (NB 1). Current definitions of a significant change and sufficient clinical evidence were therefore often described as somewhat of “a black box” (consultant 1).

### Clinical Evidence Requirements

Linked to concerns of definition were the demands and expectations of clinical evidence requirements, an area considered to be particularly disproportionate for SMEs: “*MDR has a tremendous focus on clinical evaluation, and they use these politically correct words that it’s scalable and not intended to hamper market access, blah, blah, blah. But the reality is that the requirements are not scaled. It doesn’t say for a smaller company you can do this and this. Clinical evaluation is proving the safety and clinical performance based on clinical data. That’s already a headache for large companies. It costs them millions. A small company, how are they going to do that? Personally, I haven’t got a clue”* (NB 2). Participants also expressed concerns at the continuing reliance on randomised controlled trials and “*old school compliance”* (innovator 3), that often excludes real-world data.

### Increased Workload, Costs, Delays and Need for NBs

Finally, described “*as the biggest change”* rule 11, which significantly increases the number of SMEs that need their software product assessed by a NB was considered “*time consuming… costly”* (consultant 4) and “*a nightmare for a lot of start-ups”* (consultant 1), exposing a further disadvantage for SMEs. The “*extra documents and procedures”* (consultant 1) required and unexpected delays/resources meant an investor no longer invested in “*Class III anymore… a decision based on newer regulations and the costs relating to these regulations”* (investor), highlighting a further outcome of regulatory processes.

## Communities

With regard to communities, participants identified several communities involved in the activity of medical device certification. Those most often described included innovators; health care professionals (a community considered to be particularly helpful when included in innovator teams); consultants; healthcare organisations and trade associations, a community considered to be out of reach for SMEs as “*it’s costly to join a trade association so they’re usually not there at the table”* (large company).

Communities less commonly described included commissioners, charities who often “*won’t ask for a DTAC or CE mark”* (innovator 4) and patients and the public who are often “*unaware”,* raising important questions of who existing rules and regulations seek to serve, given existing rhetoric’s around patient safety, transparency and innovation quality. For example, when asked ‘who do you think medical device certification is for?’, one innovator replied, “*well it certainly isn’t for the patients because patients really have no idea”* (innovator 9).

### Innovators

#### SMEs at a Disadvantage

Beginning with innovators, SMEs were frequently considered to be at a disadvantage by both innovators and NBs: “*they specifically mention small and medium-sized companies and innovations. Now from my experience…, I’m seriously doubting that MDR will have any beneficial effect on innovation on small and medium-sized companies”* (NB 3). Similarly, *“most of the organisations who have difficulties with it are start-ups… it’s not very start-up friendly”* (innovator 3).

Reasons for this perceived disadvantage included a lack of available “*knowledge in house”* (innovator 3); availability of “*good quality teams”* typically seen in “*larger or medium-sized companies”* (NB 3) who “*might have whole departments just for regulation”* (NB 1); and lack of previous experience within the medical device sector. As one notified body explained, “*you can’t underestimate the cultural level differences”* (NB 1) between SMEs and larger companies already familiar with the medical device landscape.

#### Cost and time

Many participants also suggested that stakeholder experiences of medical device certification processes “*depends”* (consultant 1) on availability of resources and time. Estimations of costs included “*minimum of 20,000 pounds”* (consultant 2); “*between 50 and 100,000”* (innovator 2); and “*anywhere between three and 500,000 pounds, that’s probably still conservative”* (innovator 1). Estimations for time included “*average nine to 12 months”* (innovator 3), to “*at least 2 years, minimum 2 years”* (consultant 1). However, participants also acknowledged that a lot of time is unaccounted for in most calculations particularly when considering clinical evaluation, an activity often requiring significant investor support—“*to develop a DiGA* [term used in Germany for Digital Health Applications] *our calculation is you need at least three million euros, you need to have some good investors to get three million euros if you only have an idea for a product”* (consultant 1).

### Consultants

Due to a lack of clarity and evolving regulations, many innovators were also considered to be “*heavily dependent”* (consultant 2) on “*external specialist consultants”* (innovator 2). For one participant, this was indicative of the “*catastrophe”* the medical device industry finds itself in—“*if consultants exist, it’s a sign that the industry is inefficient”* (consultant 3).

#### SMEs at a Disadvantage

However, whilst often described as “*very helpful”* (innovator 2), SMEs were again considered to be at a further disadvantage due to an over reliance on consultant support: “*my organisation is me, I would have to engage an external consultant to help, but I don’t have a huge amount of money to pay people to do that”* (innovator 6). Whilst it was considered “*certainly possible”* for an innovator to achieve medical device certification without consultancy support, “*that’s probably for more larger organisations which have a QA manager in place, a couple of people dedicated to getting their products certified”* (health authority 2). Similar concerns were also expressed by a NB—“*theoretically it’s possible but I fear for the majority of especially smaller companies, that’s not a good idea…”* (NB 2), highlighting a further disparity between the experiences of SMEs and larger companies navigating the medical device landscape.

Participants also expressed concerns at the difficulty of finding high-quality consultants e.g. “*you have the problem of finding a good one”* (NB 2). The ability to find good consultants was again considered to be “*not so easy to find as a start-up company”* (NB 3), highlighting a further contradiction for SMEs.

### Clinicians

Conversely, the inclusion of clinicians in the innovator team was frequently described as a beneficial approach. For example, “*there are a lot of practitioners in the companies we work with and that helps a lot because they actually work in the industry in which the products are being placed, they understand the concepts of efficacy and safety”* (consultant 2).

### Notified Bodies

#### Variation in Interpretation

Whilst one participant described the “*job”* of a notified body “*to show uniform alliance, uniform interpretation”* (large company), many participants including NBs themselves reported significant variation in interpretation both within and between such organisations. For example, “*you talk to different people, you get different answers”* (investor); “*there’s a huge difference between the auditors and notified bodies”* (consultant 3).

#### Difficulties Arising from Notified Bodies Being Unable to Consult

One clear division of labour expressed by participants was that NBs cannot consult. Whilst seen as frustrating by innovators, particularly with regard to clinical evidence, two NBs believed this division of labour “*should not change”* as they may otherwise lose their “*independent view”* and place “*more pressure on their resources”* (NB 3). However, some innovators acknowledged that checking clinical evaluation proposals “*is possible in the US”* (large company), an activity seen as particularly valuable in supporting the smooth functioning of regulatory processes.

#### Power Dynamics and Issues of Capacity

Reflecting possible power dynamics within the division of labour, two consultants acknowledged that innovators are often at a disadvantage when they experience variation in a NBs’ interpretation—“*as a manufacturer, you always lose”* (consultant 1); “*unfortunately the power is with the auditor… you can’t argue with an auditor, you can’t correct their understanding you just have to go through the painful process”* (consultant 2).

Furthermore, underpinning many of the concerns raised above is a perceived lack of capacity amongst NBs- “*we need to be double in size… I can serve 60% of our current clients, maybe 65”* (NB 3); “*there’s obviously a lot less notified bodies, so we are limited by how many manufacturers we can take, we are at capacity”* (NB 1). Reasons for a reduction in capacity included the: “*explosion”* of work associated with recent regulatory changes; ongoing maintenance of “*customer MDD certificates, surveyance, vigilance”* (NB 3); the significant “*learning curve”* (large company) currently being undertaken by NBs; reduction in the number of operating NBs; difficulties finding skilled workers in “*an already scarce market”* (health authority 2) as *“all notified bodies are fishing in the same pond, as are consultancy companies and the manufacturers”* (NB 3) and the “*unattractive”* (investor) process of becoming a notified body, a process described as taking “*way too long”* (NB 3). A reduction in capacity was also felt to prevent opportunities for NBs to do “*things which speak more directly to patient safety”* (health authority 1), as originally intended.

Concerns were also repeatedly expressed by both innovators and NBs that issues of capacity will worsen ahead of: “*2024 because 2024 is the latest expiry date of an MDD certificate… we are already in a crisis, the whole industry, and that crisis will only increase towards 2024 when the MDR will be mandatory. I do not have much hope… it’s not going to be a happy story”* (NB 2). Similar concerns were expressed by several participants: “*I know a lot of start-up companies are aiming to go to market in 2024. That’s not going to work because that’s when the big peak moment is coming… In our notified body, about 50% of our MDD certificates are expiring on the same day. For Europe, it’s worse, it’s 60 plus percent… That’s why I know we are about half the size of where we need to be”* (NB 3). Such concerns were believed to hold important implications for innovation safety, access and support, suggesting further contradictions between the proposed intention of medical device certification processes and the experienced reality.

#### SMEs at a Disadvantage

Finally, SMEs were perceived to be at a disadvantage in accessing already overstretched NBs because “*they* [notified bodies] *are so occupied by their normal customers, they do not need new customers. A small start-up with 10 people, you are always in one of the worst positions with a notified body and that’s killing innovation. That’s the reality”* (consultant 1). Similarly, “*what you see is the bigger companies have more notified bodies, so this whole notified body thing, hits the smallest”* (NB 2); “*if you have a huge med tech company and they would like to recertificate a thousand medical devices in the next few years, the notified body will always jump on that because they know this is a cash flow for the next 3 years”* (consultant 1).

The capacity of NBs combined with the perceived ‘unattractiveness’ of SMEs, regardless of their innovation capabilities appeared to be a further tension experienced by both innovators and NBs within existing certification processes.

### Outcome

Whilst the intended outcome of existing certification processes is medical device certification approval and/or rejection, several other unintended outcomes were also identified by participants as outlined in Table 1.

**Table 1:** Unintended outcomes of current medical device certification processes and their resulting impact

Unintended outcome	Verbatim examples	Resulting impact
Intentionally limiting the scope and functionality of innovations so they do not require medical device certification and/or fit into a lower medical device class	“*We made the decision not to go for MDR”* (innovator 9) “*We’re keeping it on purpose to a medical device class I”* (innovator 1) “*You quickly change your product, its intended use, or the way that it works to make sure that it doesn’t fall within the medical device regulations. You’ve got a less feature rich product and that is something that happens quite a lot with some companies. They tend to be frightened of the regulations so they will not develop it. They’ll circumnavigate the regulations so they don’t have to go anywhere near them because they’re terrified of how much it will cost, how long it will take and so on.”* (consultant 2)	Reduction in innovation functionality, availability, quality and safety
‘Killing’ of medical device innovation, particularly amongst SMEs	“*I’m going to be very open and frank, I think the MDR is killing innovation”* (innovator 9) “*The time it takes with a notified body and the money it takes for using the notified body, that’s killing innovation for sure… Most of the companies stopped working in the healthcare area.”* (consultant 4). “*If “You’re looking at building an ecosystem to promote innovation in health care, MDR sure isn’t helpful”* (investor) *“We’re missing out on innovation unless it’s done by a large company”* (NB3) *“True innovation is coming from small businesses; those small businesses don’t have the resources for investing in such compliance.”* (innovator 9)	Reduction in innovation frequency and opportunity, development of an ecosystem that isn’t designed to ‘promote innovation’ and withdrawal of innovators from the healthcare market
Loss of existing products	“*Some of the products that are going off market are critical. I know already myself of two products that are critical. One is that patient group had one product and it’s gone. The company just doesn’t consider the European market interesting anymore. So yes, there are products that go off markets that are going to have an effect on our healthcare”* (NB 3) “*If you look at the innovation, we are losing some kind of innovation because of these new hurdles. That’s true”* (investor)	Loss of patient critical innovations, affecting patient safety and quality of care
Loss of competitive market edge	“*Well, before typically we had companies that came to Europe because you could go to market within sort of three years. US on average was 5 years, Japan on average is 7 years. There's publications I don't invent these data. But what we have today is we are also becoming slower and more expensive*… *With our current timelines, we’re not even competitive anymore against FDA. Today, if I get approved in the US, I have access to what 350 million people? If I get approved in Europe, I get access to nothing yet because I still need to negotiate with every country*” (NB 3)	Reduction in smooth functioning of internal markets, loss of competitive market edge that may affect access to innovations that supports high level of safety and health
Creation of an unethical process	“*The world of medical devices has shifted towards larger, richer companies, it is no longer that accessible for smaller companies. Those smaller companies who are, I have to say in my view, the real innovators. Large companies do incremental innovation, they make their products a tiny bit better, but they do not come up necessarily with really new brilliant solutions. So basically, we are denying our population really serious improvements in health care. Plus we as notified bodies have to protect ourselves as well. We need to be careful with who we onboard and that is almost always in favour of established companies where we know that we can have good files. That’s why I don’t think it’s ethical. Although I’ve heard some really stupid things at the EU level already, they still claim it’s in the favour of the patients, but I don’t know what patients they’re talking about”* (NB 3)	

As a result, many participants viewed the current process of medical device certification as “*excessive”* (innovator 1), not “*ethical”* (NB3), “*not fit for purpose”* (consultant 2) and unsustainable. As suggested by one NB:

“*The world of medical devices has shifted towards larger, richer companies, it is no longer that accessible for smaller companies. Those smaller companies who are, I have to say in my view, the real innovators. Large companies do incremental innovation, they make their products a tiny bit better, but they do not come up necessarily with really new brilliant solutions. So basically, we are denying our population really serious improvements in health care… That’s why I don’t think it’s ethical. Although I’ve heard some really stupid things at the EU level already, they still claim it’s in the favour of the patients, but I don’t know what patients they’re talking about*… *There is no way this can be a success… there is not enough resource and not enough time in order to not affect innovation and not affect patient care… it will affect manufacturers, it will affect the availability of products and it will affect your patient health”* (NB 3)

### Suggestions

**Fig. 1:**
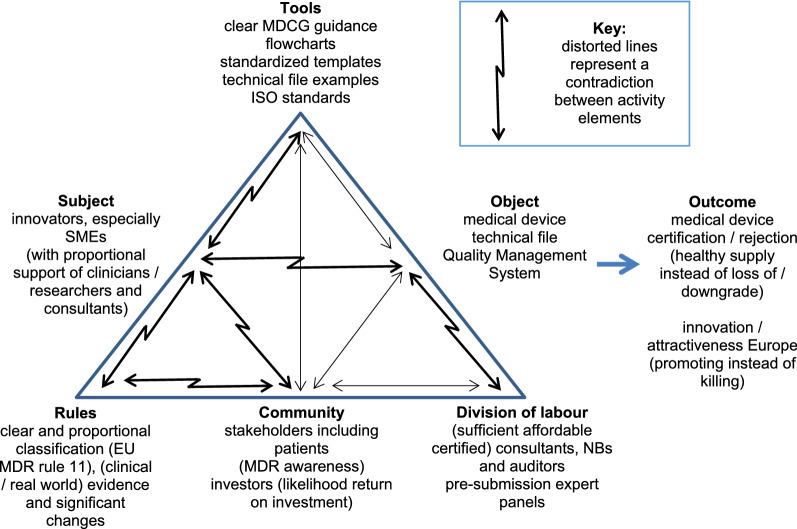
Activity framework showing contradictions between affected activity elements

Finally, Table 2 outlines participant suggested solutions to overcome some of the identified contradictions in stakeholder experiences (Fig. 1). These are grouped according to the interdependent elements of activity theory.

**Table 2:** Participant suggested solutions to address contradictions in existing medical device certification processes

Suggested solution	Verbatim example
*Rules*	
A rebalancing of proportionality	“*There has to be the right balance between the opportunities for innovation and the safety regulations… we’re balanced a little bit in the wrong way at the moment”* (investor) “*At the moment, it’s really out of balance, it’s really overshoot”* (NB 2)
Provide clearer, more harmonised laws	“*Write smarter, simpler laws with clearer language”* (large company) “*Pleading for more harmonisation within Europe on all kinds of levels”* (innovator 3) “*Ensuring the pathway is joined up with other parts of the system as well, we need to take that friction out of the market”* (NB 1)
Adopt a similar approach to regulating medicines	*“If you look at the existing regulatory framework for medicine where there are periodic assessments on batches of drugs. All of that legislation has been used for decades and we're now looking at creating brand new AI based regulatory frameworks when all we would need to do is adopt similar rules and regulations to drugs and pharma medicines production. Just periodically test these products to see whether they were still meeting the original certification and claims and evidence*” (consultant 2)
Use ISO standards only Explore regulatory sandboxes	*“Just stick with the ISO standards, and maybe have one of the quality management systems like 13,485” (innovator 9)* *“Can we create regulatory sandboxes? You would be able to engage with certain hospitals, probably regional hospitals to develop the device but also testing a device so that as a manufacturer, you get your clinical evidence in a way that meets the needs of the authorities. But the users, the clinicians or the patients get trust and much more insight into the health economic data of the use of such systems, how it's going to affect their clinical pathways, how they have to tune it, whether they will get approval of scale… The insight in those technologies is supposed to be increased through these regulatory sandboxes. The US has such a system. We have to be careful that we do not create a two-tiered system where you could forever use that sandbox and try and get on the market that way, so it should always be in my view limited in scope and time for market authorization” (large company)*
*Community*	
Improve awareness and understanding through increased communication	“*Communicating to different stakeholders clearly in a language that they understand… getting more involved in the pre-market space… and communicating in their preferred method of communication*” (NB 1) “*Getting it to their attention that there is such a thing as MDR”* (health authority 2)
Tools	
Provide easy to understand checklists/decision-making flowcharts	*“A checklist to say, if you apply for the medical device regulation in this way, here are the things that you can or must do… that would be very, very beneficial*” (innovator 2) “*A tool that can take me through my decision-making process… that would be absolutely helpful”* (innovator 8) “*Some kind of online tool to get a better understanding of what class are you and what does it mean if you’re in that class? That would be helpful”* (investor)
Provide standardised templates approved by NBs	“S*tandardisation of templates across the industry, you’ve got a level playing field then”* (consultant 2) “*If they publicly announced that they accept these kinds of templates, that will be a great first step”* (innovator 3)
Provide completed examples	“*Publishing concrete examples… like a submission database”* (consultant 3)
Monitoring service	“*Provide a monitoring service for smaller companies where you remind people to help reduce their regulatory burden. They don’t have to have that in house resource reviewing oh we have this coming up, we need to do this”* (innovator 5)
*Division of labour*	
Provide pre-submission expert panels	*“First of all, what happens, you have an expert panel… and that will be made publicly available. So everybody working in that space can consult and learn from these anonymised experiences… we’d very much like to see the system that you have in the US where you can do a pre-submission application. Can the expert panels give their comments on what we propose so that we have a bit more certainty before we start testing”* (large company)
Develop NBs that focus on lower risk medical devices only	“*If you look at where the pressure points are, they are around class I medical devices and borderline products, If you could grade notified bodies, I think you could possibly very quickly allow a number of organisations to provide that buffer for class I products, I know it’s self-certification at the moment, but I think for the borderline products to possibly get more notified bodies out there just for the lower risk medical devices… pick up the high volume low risk”* (consultant 2)
Develop a consultant marketplace	“*A trusted pool of professional advisors, that are vetted, verified, up to date with all the regulations, legal frameworks changes*… *absolutely*” (innovator 5) “*That would be helpful, it’s a no brainer, yes absolutely”* (innovator 8) “*Having a centralized list of reputable companies that don't charge excessive amounts of money, and have a proven track record working with SMEs or small innovators would be good as well*” (innovator 6)
Certify consultants	“*A licence to practice for so called experts or consultants in this area and maybe some kind of certification process to demonstrate their experience and their knowledge?”* (consultant 2)
Include medical device regulation in education	*“What is missing is at the educational level… nobody sees it when they go study as a career path…I’m not aware any learning institution where you could gain that sort of knowledge”* (consultant 3)

## Discussion

This research directly responds to previous recommendations regarding the exploration of how medical device regulations impact digital health innovation [16]. Through the innovative application of activity theory, this research advances existing knowledge and understanding by identifying contradictions across the regulatory landscape from multiple stakeholder perspectives.

### Summary of Key Findings

Whilst participants largely viewed medical device certification as necessary, clear systemic tensions exist between current *rules, tools, community* understanding, and *division of labour,* jeopardising innovation opportunities, care quality and patient outcomes. Existing rules are described as unclear and subject to unhelpful political influence. Furthermore, despite being described as the true drivers of innovation, SMEs were frequently acknowledged as being disproportionality affected by current regulatory requirements and realities. These include the higher classification (rule 11), evidence requirements which largely ignore the opportunities of real-world evidence, reliance on scarce and expensive consultant expertise, in the absence of inhouse knowledge and useful templates and examples, availability of necessary resources (time and money) and perceived undesirability from a NBs perspective [20]. Concerns of capacity amongst NBs were repeatedly raised across all stakeholders interviewed including NBs themselves, with concerns that issues of capacity may be detrimental in supporting both current and future innovations.

Following these identified tensions, participants described several intended and unintended outcomes of current medical device regulations. These included the limiting of digital innovation design, the perceived ‘killing of innovation’, loss of existing products and previously held competitive market edge. As a result, the activity of medical device certification was repeatedly described as being unfit for purpose, unsustainable, and in one case, unethical. Such findings strongly suggest that the desired aims of current medical device regulation i.e. “*to ensure the smooth functioning of the internal market… taking into account the small and medium-sized enterprises that are active in this sector”* ^(p.L 117/1)^ [16] is not yet being fully achieved. The European Commission appears to acknowledge in its draft Horizon Europe Health programme for 2023 the need for development and harmonisation of methodologies for assessing digital health technologies and the related needs to better inform innovators on the relative safety and effectiveness evidence requirements, predict market attractiveness and enable EU citizens access to person-centred digital technologies.

Interestingly innovators, NBs, health authorities and consultants shared more similarities than differences when discussing the barriers and enablers currently faced when navigating the medical device certification landscape. The only area of disparity identified was the desire for NBs to consult, particularly on clinical evaluation proposals, with NBs expressing concern that such a move would affect their independent voice, whilst many innovators felt such a proposal would be beneficial. EU MDR Article 106 seems to be provide the means to address this need by appointing expert panels.

### Strengths and Limitations

Strengths of this research include its application of activity theory as an interpretative framework, participation of 22 diverse stakeholders providing a range of perspectives, and exploration of medical device certification processes across the UK and EU supporting international relevance of findings. Data saturation (defined as the point at which no new generic themes or variations of a given theme emerged) was also achieved, with clear synergies between the barriers and enablers described by participants regardless of their position i.e. innovator, NB etc. providing further confidence in the transferability of conclusions drawn. However, the limitations of this research must also be acknowledged. This research draws on a volunteer, purposeful sample. The risk of bias associated with this sampling technique is therefore acknowledged. Finally, not all participants spoke English as their first language. Some issues of interpretation may have therefore been present. However, this risk was somewhat mitigated by the presence of a native speaking interviewer in the majority of interviews who was able to translate where required.

### Comparison with Existing Literature

The findings of this study strongly align with those reported in the EIT Health report [16], with our research providing additional insights from a health app/software perspective. Key areas of similarity include the repeated acknowledgement that ongoing issues with medical device certification processes may be preventing timely access to potentially life-saving devices; discouraging innovation; causing a substantial increase in regulatory time and expense resulting in a loss of innovative products from a previously held competitive marketplace.

Other areas of similarity include the previous acknowledgement that the regulatory landscape and accreditation process is complex, and at times confusing [8, 15, 26], with the *“*fail fast, fail often” mantra often espoused by start-ups becoming increasingly frustrated by the existing regulatory landscape [8]. As recently suggested by Ivanov et al., “*an approach is used in which regulations serve as a framework that defines the ultimate goal, but not the way to achieve it… this obliges manufactures to follow this framework but to choose the path themselves”* ^(page 44)^ [15], reflecting the variability in approaches and understanding reported in this research. The increased complexity of being aware and understanding regional variations in regulatory requirements was also frequently acknowledged in this research, further supporting existing literature that calls for increased harmonization and international standardization [15, 26].

Unique contributions, not previously discussed in academic research concerning medical device certification, include the identification of more harmonization than disparity between the experiences and perceptions of interviewed innovators, NBs, consultants, investors and health authorities. EU MDR rule 11 on classification of software is largely found to be disproportional and especially counterproductive for SMEs who are acknowledged to be the true drivers of innovation. The quotes of participants add richness to the EIT Health findings and paint the current reality in rather harsh terms. A reality that was described more firmly than in EIT Health’s report to result in downgrades, unique products taken off the market, investors pulling out and Europe’s attractiveness eroding, with a further decline looming in 2024 when MDD certifications expire. All of the above negatively affect patient’ access and health outcomes. What is considered sufficient evidence, why not also include real-world evidence and what is a proportional approach to a significant change and related need for further evidence are questions that are addressed as urgently needing answers. The multidisciplinary focus of this research provides additional insights, experiences and suggestions that were previously unavailable in existing knowledge and understanding. In particular, suggestions on what is expected effective tooling add to the EIT Health findings. EU MDR Article 106 (pre-submission expert panels) may provide another useful suggestion.

### Implications

The implications of this research are clear. Firstly, more needs to be done to ensure clarity of understanding, and universal interpretation of medical device certification processes. As suggested in the EIT report, this does not necessarily mean less regulation, but rather implementing easier, clearer, and faster processes that do not hinder innovation production and access as currently experienced. Participant suggestions of developing freely available templates and completed examples that are endorsed by NBs may help facilitate this clarity of understanding, whilst simultaneously streamlining the medical device certification processes for all involved. Participant suggested templates included those produced by Open Regulatory and the Johner Institute.

Secondly, attention needs to be paid to enhancing the sustainability of existing certification processes. Participants including NBs and innovators perceived NBs to already be at capacity, an issue anticipated to only worsen given the expiration of MDD certification. Participant suggestions of developing NBs that focus on lower risk medical devices only which could be an answer to making rule 11 more proportionate and accelerating the process for becoming a NB may help address this concern, as could incorporating medical device certification processes into existing undergraduate/postgraduate courses to further support the limited skilled workforce currently available.

Thirdly, critical consideration needs to be paid to the original intention of medical device certification and who it seeks to serve. Currently, there seems to be a disproportionate aversion to risk [8], with some patients and the public experiencing a loss of innovation as opposed to a promised ‘high level of protection of health’^(1.117/1)^ [16]. Whilst several participants perceived the introduction of more stringent regulations (in particular rule 11) as a direct response to previous failings such as the PIP breast implant scandal, questions must be asked of whether the revised regulations would prevent a similar scandal, or if the risk of a repeat scandal has been replaced by other unintended consequences such as adjusting the intended use of innovations to avoid medical certification regulations altogether.

Co-developing international consensus on what constitutes a significant change, sufficient clinical evidence for certification, reimbursement and adoption purposes would also be highly beneficial [8, 26], as would acknowledging more innovative research designs [27–29], that fit the ever-evolving characteristics of software and usability testing in real-world setting [5, 26]. As recently suggested by Khadjersai et al., where the level of evidence differs across countries, strong justification for such variation will be required [26].

Finally, attention needs to be paid in identifying practical ways to meaningfully support SMEs in medical device certification given their repeatedly acknowledged disadvantage under current regulatory rules and requirements. Only then can the desired aim of current medical device regulations be fully realised. Failure to implement such changes may lead to an undesirable stifling of innovation that inhibits, as opposed to accelerates digital innovation.

## Conclusion

The original intention of establishing a robust and sustainable regulatory framework that ensures a high level of safety and health whilst supporting innovation and SMEs is not yet being realized. Existing processes are described as overly complex, subject to variable interpretation that requires significant resources with limited apparent benefits. Our findings suggest more must be done to ensure not just the sustainability of complex MDR processes, including accelerating processes for becoming a NB, but also an improvement in clarity and understanding of exactly what is required of developers. Given that developers are often disadvantaged through a lack of technical expertise, MDR open templates were repeatedly cited as a practical suggestion, as were incorporating medical device certification processes into existing undergraduate/postgraduate courses. In the absence of acknowledgement or action, it is likely that medical device certification processes may continue to remain unfit for purpose and unsustainable.

## Supplementary Information

Below is the link to the electronic supplementary material.

Supplementary file1 (DOCX 19 kb)
