# Supplementary material for: Navigating Medical Device Certification: A Qualitative Exploration of Barriers and Enablers Amongst Innovators, Notified Bodies and Other Stakeholders
Source: Ther Innov Regul Sci. 2022 Oct 4;57(2):238–50. doi: 10.1007/s43441-022-00463-4 (PMC9531632; doi:10.1007/s43441-022-00463-4)
Supplement: Supplementary file 1 — Supplementary file1 (DOCX 19 kb) [file 43441_2022_463_MOESM1_ESM.docx]

Semi-structured interview schedule – Innovators

# Background:

The world of Medical Device Certification can be extremely complex and difficult to navigate. At ORCHA, we are keen to understand the experiences and perceptions of digital health innovators navigating the medical device certification process, particularly for Software as Medical Devices (SAMD).

We would like to speak to digital health innovators who have developed native health-apps and have either i) successfully applied and achieved Medical Device certification ii) have applied for Medical Device certification but were unsuccessful, or iii) are currently, or soon to be applying for Medical Device certification for their digital health innovation.

There are no right or wrong answers so please be honest in your responses. All content will be anonymised.

Do you have any questions before we start?

Do you give your consent to take part?

# Questions - Innovators

Demographics

1. Please describe your role and where you are comfortable, your gender.
2. Are you a healthcare professional?
3. What stage of business development is your digital health technology currently at? We have a couple of options that I will read out to you. If you have more than one product or are part of more than one company, please answer only in relation to the technology with the highest level of development.

- Stage 0 – A digital health company that has not yet developed a digital health technology
- Stage 1 - A digital health company with a well-developed idea or early digital health technology(s), or a prototype which requires testing or evaluating with end-users
- Stage 2 - A digital health company with established digital health technology(s) that has completed initial testing and is now looking for additional testing and/or larger scale implementation of the product
- Stage 3 - A digital health company with established digital health technology(s) looking to expand to different markets or increase sales within the current market or considering spin-outs/additions to current product range

1. What tier of the [NICE Evidence Standards Framework](https://www.nice.org.uk/corporate/ecd7/resources/evidence-standards-framework-for-digital-health-technologies-pdf-1124017457605) do you consider your digital health technology to be? Again, we have a couple of options that I will read out to you.

- Tier A - Digital health technologies with potential system benefits but no direct user benefits
- Tier B - Digital health technologies that help users to understand healthy living and illnesses but are unlikely to have measurable user outcomes.
- Tier C - Digital health technologies for preventing, diagnosing and managing diseases. They may be used alongside other treatments and will likely have measurable user benefits. DHTs in the treat, active monitoring, calculate or diagnose categories will likely be CE marked medical devices
- Don’t know

1. Have you successfully undertaken a conformity assessment and achieved the UKCA or EU CE mark?

Experiences

1. At what point in your innovation journey did you become aware that your digital health technology would require medical device certification?

Probes:

- What resources/sources of information did you use to reach your decision?
- Do you think there’s any ambiguity in the NICE Evidence Standard tier framework? If so/not, why?

1. How would you describe your experience of applying for medical device certification?

Probes:

- Were there any aspects that you found particularly difficult or challenging?
- What, if anything, did you do to overcome any of the challenges you faced?
- Were any parts of the process particularly easy?
- How long did the process take/how long do you think it will take?
- If happy, please share an approximate cost your organisation encountered or expects to encounter to achieve medical device certification

1. Which roles within your organisation were involved in navigating the medical device certification process? Please describe all those involved

Probe:

- Why were these roles chosen?

1. Did your organisation receive any external support when applying for medical device certification?

Probes:

- Who did you receive/plan to receive support from?
- What did they do?
- Did you receive external support for any of the four following areas: risk management, data privacy and security, clinical evidence, usability and accessibility
- How much would you say it cost your organisation to engage with external support?

1. Could anything be done to improve your experience of navigating the medical device certification process? If so, please tell us more.

Probe:

- Were there any pain points (organisation, time, cost, lack of understanding, paperwork?)

1. Could a technological solution, including a step by step guide be of use? What benefits do you believe this may have provided to you, or could provide to you going forward?
2. Would you be interested in taking part in a future co-design workshop or interview about your experiences?

# Questions – Notified Bodies

1. Please could you describe a little bit about your background and role?

2. Based on your experiences, how do you think innovators of health software find the process of applying for medical device certification? Probe: what if anything is working particularly well? What if anything could be improved?

3. At what point in the innovation journey do you think innovators typically become aware that their digital health technology might require medical device certification? Probe: is this early enough? When should they become aware and why?

4. How long you think it takes innovators on average to navigate the medical device process?

5. What if anything could be done to help improve the process for both regulators and innovators applying for medical device certification? Probe: what would you recommend to SMEs seeking to navigate the medical device process?

6. What if any resources/websites would you recommend to facilitate the process of establishing whether a digital health technology is a medical device or not?

# 
